# Co-Infection of Chicken Infectious Anemia Virus and Fowl Adenovirus Serotype E8b Increases Mortality in Chickens

**DOI:** 10.3390/v17050620

**Published:** 2025-04-26

**Authors:** Lin Liu, Wenming Gao, Jingjing Chang, Jingrui Liu, Zongmei Huang, Wenjie Sun, Yapeng Song, Xinsheng Li

**Affiliations:** College of Veterinary Medicine, Henan Agricultural University, Zhengzhou 450002, China; 15638870010@163.com (L.L.); 15803826941@126.com (W.G.); c2640460041@163.com (J.C.); l2639402115@outlook.com (J.L.); ndkjchuang@163.com (Z.H.); 18860363109@163.com (W.S.)

**Keywords:** fowl adenovirus serotype E8b, chicken infectious anemia virus, co-infection, pathogenicity

## Abstract

The chicken infectious anemia virus (CIAV) and fowl adenovirus serotype E8b (FAdV E8b) are pathogens that cause aplastic anemia and inclusion body hepatitis (IBH) in chickens, respectively. The co-infection of CIAV and FAdV E8b poses a significant threat to poultry health, potentially worsening clinical symptoms and increasing mortality rates. This study aimed to explore the combined pathogenic effects of FAdV E8b and CIAV co-infection on one-day-old specific pathogen-free (SPF) chickens. The results showed that co-infection led to significantly higher clinical scores and mortality rates compared to FAdV E8b infection alone. Additionally, there were different tissue distribution patterns for FAdV E8b between the single infection and co-infection groups, indicating potential changes in viral tropism. Biochemical analysis revealed elevated markers of liver and/or muscle damage in both the FAdV E8b infection group and the co-infection group, consistent with the viral infection process. These findings suggest that co-infection with FAdV E8b and CIAV can intensify clinical signs and mortality, and may potentially alter viral replication and tissue tropism in chickens. This study establishes a foundation for future investigations into the underlying mechanisms governing the interaction between CIAV and FAdV E8b during co-infection.

## 1. Introduction

Chicken infectious anemia virus (CIAV) has recently been classified as a member of the Gyrovirus genus within the *Anelloviridae* family by the International Committee on Taxonomy of Viruses (ICTV) [1]. The primary clinical symptom of CIAV infection is aplastic anemia. CIAV infection disrupts the immune system by directly damaging crucial tissues responsible for blood cell production (hematopoietic tissue) and lymphocyte development (thymus) [2,3]. Its key impacts on non-specific (innate) immunity in chickens are lymphoid tissue depletion, impaired macrophage function, reduced interferon production, and general immunosuppression [4,5]. CIAV’s high PCR-positive rates (46.67% to 81.25%) are often disregarded due to its low direct mortality, but the virus’s role in facilitating secondary infections is a major contributor to overall morbidity and mortality [6,7,8].

Fowl adenoviruses (FAdVs) belong to the genus Aviadenovirus within the Adenoviridae family. FAdVs are classified into 12 serotypes (1 to 8a and 8b to 11) within five species (A to E) based on serology analyses and genome characterization [9]. Since 2015, FAdV serotypes 4, 8b, and 11 have emerged as the most prevalent strains, showing high infection and mortality rates in chicken flocks [10,11,12]. FAdV can cause inclusion body hepatitis (IBH), hepatitis hydropericardium syndrome (HHS), and gizzard erosion and ulceration. Different serotypes of FAdV cause different symptoms; serotypes 2, 8a, 8b, and 11 are the main serotypes causing IBH [13,14,15]. Additionally, the spread of FAdV has accelerated the virus’s evolution, resulting in increased instances of genetic recombination. The natural mutant HN1472 employed in the study resulted in significant field mortality [16]. Variant HN1472 is a recombination strain, primarily composed of the genome of FAdV E8b, with the fiber gene originating from FAdV E8a.

CIAV-infected chickens become immunosuppressed, which increases their susceptibility to viral, bacterial, or fungal infections [17,18,19,20], even though CIA is often considered asymptomatic in adult chickens [21]. While most FAdV strains act as opportunistic pathogens, FAdV variant HN1472 has been observed to cause mortality in 1-day-old SPF chickens [16]. Co-infections of CIAV with FAdV and other viruses can lead to worsened clinical symptoms and higher mortality rates [17,19,22], resulting in significant economic losses in the poultry industry. This study aimed to assess the clinical lesions, histopathological changes, virus load of tissues, and viral shedding patterns in SPF chickens infected with CIAV HN1412 strain and/or FAdV E8b variant HN1472 at day 1 of age. Additionally, changes in biochemical indicators were monitored to better understand the dynamics of the co-infection. The research sought to study how the interaction between host and pathogens, and the effects of co-infection on chickens, can inform future strategies for prevention and treatment.

## 2. Materials and Methods

### 2.1. Viruses and SPF Chicken Embryo Eggs

The CIAV strain HN2021-1412 (GenBank: MZ369153.1) was isolated in Henan Province in 2021 and subsequently cultured in SPF chicken embryo yolk sac with an embryo infectious dose (EID_50_) of 10^5.0^/0.2 mL. The FAdV E8b HN1472 strain (GenBank: OR975470.1), also isolated in Henan Province in 2021, was cultured in chicken Leghorn male hepatocellular (LMH, ATCC CRL-2117) cells with a 50% tissue culture infectious dose (TCID_50_) of 10^5.8^TCID_50_/0.1 mL. Both strains were isolated and subsequently stored in our laboratory. The SPF chicken embryo eggs were procured from Beijing Boehringer Ingelheim Vital Biotechnology Co., Ltd. (Beijing, China). The SPF chickens were subjected to humane procedures and maintained in accordance with protocols approved by the HENAU Animal Ethics Committees (HNND2023032218).

### 2.2. Animal and Experimental Design

110 one-day-old specific pathogen-free (SPF) chickens were randomly assigned to one of four groups and kept separately in negative pressure isolators. Twenty birds in the negative control group were administered 0.3 mL of phosphate-buffered saline (PBS). The sterile PBS was used to dilute the relative virus to the target volume. The group infected with CIAV consisted of 30 birds inoculated with 0.3 mL of the HN1412 strain, which contains 10^5.0^ EID_50_ CIAV, via intramuscular injection. Similarly, the group infected with FAdV E8b, comprised of 30 birds, received an intramuscular injection of 0.3 mL HN1472 strain, which contains 10^5.8^ TCID_50_ FAdV E8b. Additionally, the co-infection group included 30 birds that were co-inoculated with both 10^5.0^ EID_50_ of the CIAV HN1412 strain (0.2 mL) and 10^5.8^ TCID_50_ of the FAdV E8b HN1472 strain (0.1 mL) via intramuscular injection. FAdV E8b and CIAV were inoculated separately into each infected chicken, rather than being mixed. In contrast, the chicken in the control group received an inoculation of 0.3 mL of phosphate-buffered saline (PBS) in a similar manner. The animals were provided ad libitum access to feed throughout the 21-day observation period.

### 2.3. Sample Collection and Clinical Signs Monitoring

To quantify viral shedding, oral and cloacal swabs were collected from each group at 1, 3-, 7-, 14-, and 21-days post-infection (dpi). Furthermore, three chickens from each group were euthanized at the same time intervals as swabs sampling (e.g., 3-, 7-, and 14-days post-infection), and tissue samples (liver, lung, bone marrow, thymus, kidney, duodenum, cecum, and rectum) were collected for real-time PCR analysis to determine viral load. The tissues were subsequently fixed in 10% neutral buffered formalin for future histopathological examination. The presence and severity of clinical signs were evaluated using a scoring system with a range of 0 to 4, designed to assess the extent of the symptoms observed. A score of 0 indicated the absence of symptoms, 0 for normal, 1 for mild depression, 2 for severe depression, 3 for paralysis/prostration, and 4 for death [23]. The occurrence and severity of clinical signs were monitored and scored throughout the infection. Serum samples were obtained to measure biochemical indices after infection.

### 2.4. Histopathological Analysis

The tissue samples fixed in 10% neutral formalin were sectioned into 4 4-micrometer (μm) sections and stained with hematoxylin and eosin (HE). Subsequently, the lesions of the sections were observed under a light microscope and scored according to the severity of the histopathological changes. Score 1 (mild) represents minor pathology, such as a small amount of inflammatory cell infiltration. Score 2 (moderate) represents focal necrosis of hepatocytes, swelling of renal tubular epithelial cells. Score 3 (severe) represents a large area of hepatocellular necrosis, focal necrosis of hepatocytes, presence of intranuclear inclusion bodies (INIB), degeneration, and necrosis of renal tubular epithelial cells.

### 2.5. Viral Load Quantification with Real-Time PCR

The viral DNA was extracted from tissues and swabs using the FastPure Viral DNA/RNA Mini Kit Pro (Vazyme, Nanjing, China) following the manufacturer’s protocol. The extracted DNA was then measured using real-time PCR to quantify the viral load. Specific primers (see Table 1) were used for PCR amplification to detect the CIAV viral capsid protein 3 (VP3) and FAdV E8b hexon genes as designed before [16,24]. The resulting fragments were ligated into the pMD19-T vector (Takara, Japan) for future use. Standard curves were established and optimized to ensure accurate virus detection. The real-time PCR reaction consisted of an initial denaturation step at 95 °C for 2 min, followed by 40 cycles at 95 °C for 5 s and 55 °C for 30 s. The fluorescence data were read during the 55 °C step, and the final extension step was at 72 °C for 2 min. The reaction volumes were 20 µL per well. The real-time PCR assay was performed using the LineGene 9600Plus system (BIOER, Hangzhou, China).

### 2.6. Biochemical Assays

Serum samples were subjected to analysis using the SMT-120VP chemistry analyzer (Seamaty, Chengdu, China) in order to ascertain the levels of gamma-glutamyl transferase (GGT), total bile acid (TBA), lipase (LPS), urea, aspartic aminotransferase (AST), alanine aminotransferase (ALT), lactate dehydrogenase (LDH) and hematocrit (Hct).

### 2.7. Statistical Analysis

Statistical analyses were performed using GraphPad Prism version 9.0.2 (GraphPad Software Inc., San Diego, CA, USA) to evaluate both the real-time PCR data and biochemical measurements (GGT, TBA, LPS, urea, AST, ALT, LDH, and Hct). A two-way analysis of variance (ANOVA) was employed to assess the effects of the experimental factors on the measured parameters. Differences were considered statistically significant at *p* < 0.05.

## 3. Results

### 3.1. Clinical Signs

**Figure 1:**
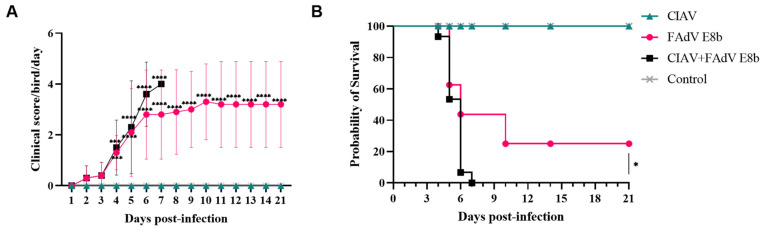
The chicken’s clinical scores and survival rates in different groups. (**A**) The clinical scores in the infected and the control groups were shown from 1 dpi to 21 dpi. Value indicated the mean clinical score per group per day. (**B**) The percentage survival of chickens is shown from 1 dpi to 21 dpi. Survival of FAdV E8b-infected cells is significant for the co-infected group. CIAV-infected group is marked in cyan triangle, FAdV E8b-infected group is marked in magenta circle, CIAV and FAdV E8b co-infection group is marked in black square, the control group is marked in gray cross. *, significant difference (*p* < 0.05); ***, highly significant difference (*p* < 0.001); ****, extremely significant difference (*p* < 0.0001).

**Figure 2:**
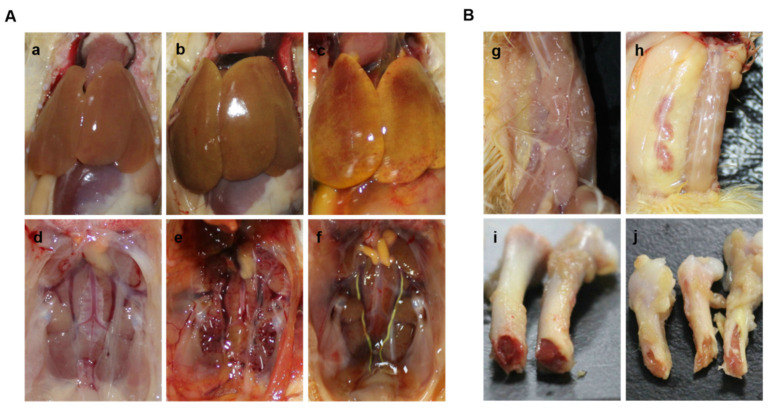
The gross lesions in the control and infected groups. (**A**) The liver and kidney of chickens in the control group (**a**,**d**), FAdV E8b-infected group (**b**,**e**), and co-infected group (**c**,**f**) at 3 dpi. (**B**) The thymus and bone marrow of chickens in the control group (**g**,**i**) and CIAV-infected group (**h**,**j**) at 21 dpi.

Significant clinical symptoms, including lethargy and feed intake, were observed in both the FAdV E8b HN1472-infected group and the co-infected group starting from 2 days post-infection (dpi). Both groups exhibited the typical symptoms of IBH and received high clinical scores (Figure 1A). The control group did not demonstrate any clinical symptoms throughout the experiment. The chickens infected with CIAV and the control group had normal appearance and no clinical symptoms, while the chickens in the 8b infection group and the co-infection group showed clinical symptoms such as depression, lethargy, and decreased feed intake. Three chickens in each group were randomly selected for euthanasia, and autopsies were performed to observe organ lesions. Classic IBH symptoms of chickens were observed in chickens infected with both FAdV E8b and co-infected with other pathogen groups at 3 dpi, including enlarged, yellow, and hemorrhagic livers, as well as kidney hemorrhage and swelling (Figure 2A). There are slight lesions in the intestines. The chickens from the CIAV-infected group displayed the hallmarks of anemia, including thymus atrophy and bone marrow yellowing, at 21 dpi (Figure 2B).

### 3.2. Mortality Rate

The chickens in the experimental groups had different degrees of death. Chicken in the co-infected group died at 4–7 dpi, and the mortality rate reached 100%. The co-infected group demonstrated a markedly elevated mortality rate in comparison to the FAdV E8b HN1472-infected group. By 7 dpi, all chickens in the co-infected group had succumbed to the infection, while the mortality rate in the FAdV E8b HN1472-infected group reached 80% and remained stable throughout the observation period (Figure 1B). In contrast, the CIAV HN1412-infected group and the negative control group showed no mortality throughout the experiment, as illustrated in Figure 1A,B. These findings indicate that co-infection with FAdV E8b and CIAV results in more severe clinical signs and significantly higher mortality compared to infection with FAdV E8b alone.

### 3.3. Histopathology Changes of SPF Chickens

**Figure 3:**
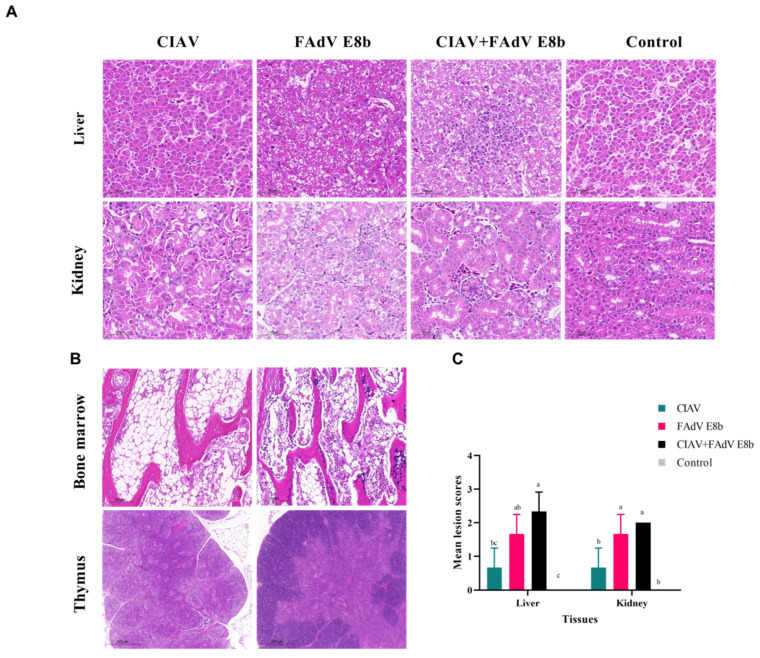
Histopathological changes of tissues in each group. (**A**) Representative image of hematoxylin and eosin-stained liver and kidney sections from chickens in CIAV, FAdV E8b, co-infection, and control group, respectively, at 3 dpi. Magnification: ×400. (**B**) Histopathological changes of bone marrow and thymus in CIAV-infected chickens (**left**) and the control group chickens (**right**) at 21 dpi. Magnification: ×50 and ×200, respectively. (**C**) Mean lesion scores in these organs at 3 dpi (*n* = 3). ^a–c^ Bars with no common superscript are significantly different (*p* < 0.05).

The results of the histopathological analysis showed that the FAdV E8b-infected and co-infected groups exhibited extensive pathological damage to the liver and kidney, which was more severe in the co-infected groups (Figure 3). No obvious lesions were observed in the control group. In the FAdV-only and co-infection group, chickens showed hepatocellular necrosis, INIB, lymphocyte reduction in the thymus, and degeneration and necrosis of renal tubular epithelial cells [25].

The CIAV-infected group displayed distinctive alterations in the bone marrow and thymus at 21 dpi (Figure 3B). There was a reduction in the size of the thymus cortex, resembling the medullary zone. A microscopic examination revealed the presence of pale yellow and fat-like marrow tissue, accompanied by a reduction in the number of hematopoietic cells and an increase in fat cells. In contrast, the control group exhibited no notable histopathological alterations in their tissues.

### 3.4. Quantification of Tissues’ Viral DNA Using Real-Time PCR

**Figure 4:**
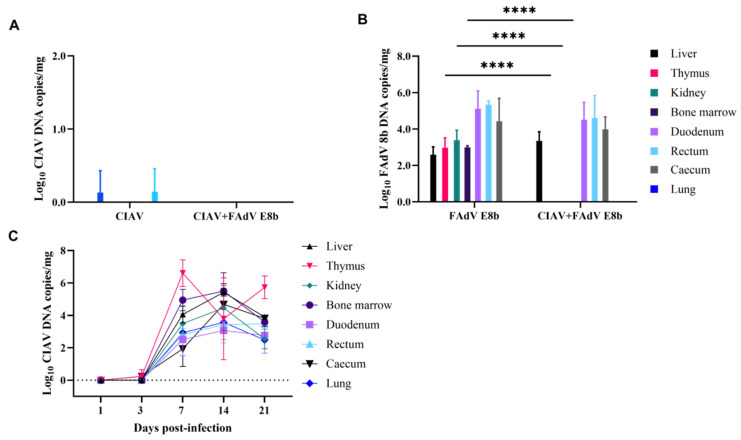
Viral loads of chicken tissues in infected groups. (**A**,**B**) The viral loads of chickens (n = 3) infected with CIAV and/or FAdV E8b were measured using real-time PCR at 3 dpi. (**C**) Changes in viral load in CIAV-infected chickens were monitored by real-time PCR from 1 dpi to 21 dpi. ****, extremely significant difference (*p* < 0.0001).

To assess viral load in diverse tissues at 3 dpi, the viral copy numbers were quantified using established real-time PCR assays specific for FAdV E8b and CIAV (Figure 4A,B). The lowest levels of CIAV were identified in the thymus and cecum of the CIAV-infected group (Figure 4A). However, the absence of significant discrepancies in viral concentrations between groups in the thymus and cecum indicates that this alteration may have minimal implications. It was unexpected that CIAV was undetectable in any tissue examined from the co-infected group at 3 dpi. The finding suggests the potential for a change in CIAV’s tissue tropism due to co-infection with FAdV E8b.

In the group infected with FAdV E8b (Figure 4B), the virus was detectable in all tissues except the lung (which was not included in the analysis). In the groups of chickens co-infected, there was no significant difference in the level of FAdV E8b viral load in the liver, duodenum, and rectum when compared with the group of chickens that had been infected with FAdV E8b alone. It is noteworthy that the FAdV E8b virus was not detected in the thymus, kidney, or bone marrow of co-infected chickens.

### 3.5. Dynamics of CIAV Tissue Distribution

The initial detection of CIAV was observed at low levels in the thymus and caecum 3 dpi. However, by 7 dpi, CIAV was detected in all tissues examined (including liver, thymus, kidney, bone marrow, duodenum, rectum, caecum, and lung), with particularly high levels (reaching 4 × 10^4^ copies/mg) observed in the liver, thymus, and bone marrow at 14 and 21 dpi (Figure 4C). The results illustrate that CIAV infection results in significant and prolonged viremia.

### 3.6. Shedding Patterns of FAdV E8b and CIAV

**Figure 5:**
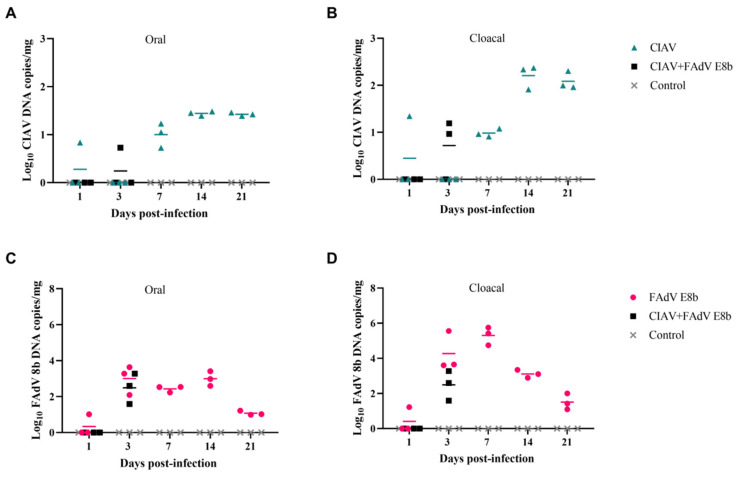
The viral shedding of chickens and the dynamic changes after being infected. The viral shedding of oral (**A**) and cloacal (**B**) from the infected and co-infected groups (n = 3) at different time points was determined by real-time PCR. Additionally, the oral (**C**) and cloacal (**D**) viral shedding of FAdV E8b-only and co-infected chickens at different time points were determined by real-time PCR.

To investigate the shedding of FAdV E8b and CIAV viruses, oral and cloacal swabs of chickens were collected from chickens in the infected and control groups during the 21-day observation period. Chickens in the CIAV-infected group showed no clinical signs or mortality throughout the experiment. CIAV was initially detected at low levels in oral and cloacal swabs at 1 dpi, but gradually increased to peak at approximately 2 × 10^4^ copies/mg at 14 and 21 dpi. There were no significant differences in the amount of CIAV shed (oral and cloacal) between the CIAV-infected and co-infected groups (Figure 5A,B) at 1 or 3 dpi. Similarly, no significant difference was found between the FAdV E8b-infected group and the co-infected group at 1 or 3 dpi (Figure 5C,D). Strikingly, the co-infection group was able to detect the excretion of CIAV virus in both oral and cloacal swabs on 3 dpi, whereas the CIAV infection group did not. These findings suggest that co-infection may affect the replication dynamics of CIAV.

### 3.7. Biochemical Maker Levels

**Figure 6:**
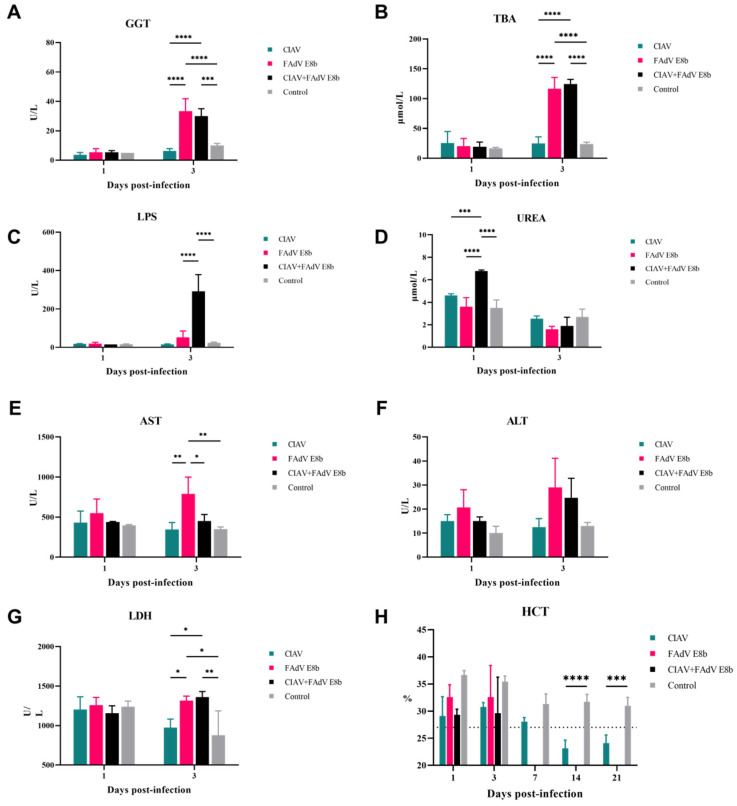
Biochemical index levels of serum in infected groups. Chickens (n = 3) were inoculated with FAdV-E8b HN1472 and/or CIAV HN1412, and the serum levels of various biochemical indices were measured. (**A**–**H**) The serum GGT, TBA, LPS, Urea, AST, ALT, LDH level and the blood Hct were measured and analyzed. CIAV-infected group is marked in cyan, FAdV E8b-infected group is marked in magenta, CIAV and FAdV E8b co-infection group is marked in black, the control group is marked in gray. *, significant difference (*p* < 0.05); **, very significant difference (*p* < 0.01); ***, highly significant difference (*p* < 0.001); ****, extremely significant difference (*p* < 0.0001).

Serum samples at 3 dpi were analyzed to assess the effect of FAdV E8b and CIAV infection on various biochemical markers (Figure 6). The serum TBA and LPS activity levels in the FAdV E8b-infected and the co-infected groups displayed a similar pattern of increase to GGT (*p* < 0.0001) (Figure 6A–C). The results suggest potential liver damage and further support the potential liver dysfunction caused by FAdV E8b infection. Chickens in the CIAV-infected group showed typical anemia symptoms at 14 dpi, with Hct values less than 27%, while the Hct values of chickens in the control group remained above 30% (Figure 6H). Serum urea levels and AST and LDH levels were significantly elevated only in the co-infected group compared to the other groups (Figure 6D,E,G), suggesting renal impairment and possible muscle or liver damage induced by co-infection. No noteworthy differences in ALT levels were noted between groups at 1 or 3 dpi (Figure 6F).

## 4. Discussion

The increasing prevalence of FAdVs in poultry farms, often accompanied by mixed infections with immunosuppressive pathogens, is becoming more common, causing significant economic losses to the poultry farming industry [12,26]. CIAV infection could interfere with the development of immune organs and inhibit the immune response of chickens, while all these effects similarly provided opportunities for FAdV to invade the chickens and then rapidly proliferate in vivo [27]. This study investigated the interaction between CIAV and a specific FAdV-8b variant (strain HN1472) using a co-infection model in SPF chickens. Clinical signs, mortality, gross lesions, histopathology, tissue viral loads, oral and cloacal viral shedding, and biochemical markers were evaluated. This study simulated the co-infection of FAdV-8 and CIAV in SPF chickens, revealing a synergistic pathogenicity between CIAV and FAdV-8b.

As previously reported, the pathogenicity of co-infection of FAdV-4 and a significant avian disease pathogen that has an immunosuppressive effect was higher than that of singular infection [17,28]. In our study, the co-infection with CIAV and FAdV E8b resulted in significantly more severe clinical signs, higher mortality (100% at 7 dpi), and a broader range of lesions observed in the liver, thymus, duodenum, rectum, and caecum than FAdV E8b infection alone [16]. This is consistent with broader patterns of viral synergism observed in poultry co-infections, while also highlighting distinct pathogenic dynamics. Also, the viral shedding of FAdV E8b could be detected in co-infection chickens but not in CIAV-infected chickens. The number of three chickens with significant differences in analysis may be limited. Different species or serotypes of FAdV exhibit varying degrees of pathogenicity in chickens [27,29]. The rapid mortality observed in this study surpasses the mortality rates reported in previous reports. This finding may suggest that the immunosuppressive effects of CIAV facilitate the proliferation of secondary pathogens, leading to variations in pathogenicity. The current research results show that there is a significant influence on the replication of FAdV E8b throughout the whole process of CIAV infection.

In this study, FAdV E8b and CIAV could be detected in the tissue samples of chicken, which was in accordance with existing research results. Notably, FAdV E8b was found in the bone marrow, thymus, and kidneys of chickens in the FAdV E8b-infected group, but not in these organs of co-infected chickens at 3 dpi. This difference in tissue distribution suggests that CIAV infection might affect FAdV E8b invasion of central immune organs, potentially impacting viral copy numbers [30,31]. The co-infection of FAdV E8b and immunosuppressive pathogens demonstrated a synergistic effect rather than an immunosuppressive effect, facilitating the action of FAdV E8b, but the real mechanism is still unclear [32,33].

We evaluated various biochemical markers to assess potential organ damage. A decrease in Hct values was observed in CIAV-infected chickens, which aligns with the characteristic clinical manifestations of CIAV infection, confirming successful CIAV induction in the chickens [34]. Elevated GGT and TBA levels in the co-infected and FAdV-8b-infected groups suggest that the FAdV-8b HN1472 variant can induce liver and kidney injury. AST exists in hepatocytes and is released into the blood during early cell degeneration [35]. Notably, AST levels were significantly elevated only in the FAdV-8b-infected group, with no significant alteration observed in the co-infected group. This observation aligns with the tissue viral load results, potentially indicating that the tropism of CIAV for lymphoid tissues hinders FAdV-8b’s ability to invade tissues in the condition of co-infection [36,37]. The co-infection group exhibited significantly elevated lipase levels compared to the single infection group, which was caused by the reduced lipase clearance, and the studies have shown that liver and kidney dysfunction and intestinal factors may play a role in the observed differences [38].

Based on our study, there may be a combined effect between CIAV strain HN1412 and FAdV-8b variant HN1472, which could potentially increase the virulence of FAdV-8b. This highlights the importance of recognizing and managing co-infections in poultry flocks. The complex nature of disease development emphasizes the need for a thorough understanding of the interaction between CIAV and FAdV-8b variants to develop more effective prevention and treatment strategies in the future [39,40,41].

## Figures and Tables

**Table 1:** The sequence of real-time PCR primers for detecting CIAV and FAdV E8b.

Primers	Sequence of Primers
CIAV-F	5′-CGTTGGAAACCCCTCACTG-3′
CIAV-R	5′-CCTCAAGTCCGGCACATTC-3′
CIAV-P	5′-FAM-CCAGTGCTTTCTGAATTGTCCGCAGTTGC-TAMRA-3′
8b-F	5′-TAGACACCACCGCACAGAAATAC-3′
8b-R	5′-TGCCTGACCGTTCGGAGTT-3′
8b-P	5′-FAM-CCAACTACATCGGGTTCCGTGACAAT-TAMRA-3′

## Data Availability

The original contributions presented in this study are included in the article. Further inquiries can be directed to the corresponding author(s).
